# Cover Crops and Fertilization Alter Nitrogen Loss in Organic and Conventional Conservation Agriculture Systems

**DOI:** 10.3389/fpls.2017.02260

**Published:** 2018-01-22

**Authors:** Rebecca E. Shelton, Krista L. Jacobsen, Rebecca L. McCulley

**Affiliations:** ^1^Department of Plant and Soil Sciences, University of Kentucky, Lexington, KY, United States; ^2^Department of Horticulture, University of Kentucky, Lexington, KY, United States

**Keywords:** ammonia volatilization, conservation agriculture, cover crops, nitrate leaching, nitrous oxide emissions

## Abstract

Agroecosystem nitrogen (N) loss produces greenhouse gases, induces eutrophication, and is costly for farmers; therefore, conservation agricultural management practices aimed at reducing N loss are increasingly adopted. However, the ecosystem consequences of these practices have not been well-studied. We quantified N loss via leaching, NH_3_ volatilization, N_2_O emissions, and N retention in plant and soil pools of corn conservation agroecosystems in Kentucky, USA. Three systems were evaluated: (1) an unfertilized, organic system with cover crops hairy vetch (*Vicia villosa*), winter wheat (*Triticum aestivum*), or a mix of the two (bi-culture); (2) an organic system with a hairy vetch cover crop employing three fertilization schemes (0 N, organic N, or a fertilizer N-credit approach); and (3) a conventional system with a winter wheat cover crop and three fertilization schemes (0 N, urea N, or organic N). In the unfertilized organic system, cover crop species affected NO_3_-N leaching (vetch > bi-culture > wheat) and N_2_O-N emissions and yield during corn growth (vetch, bi-culture > wheat). Fertilization increased soil inorganic N, gaseous N loss, N leaching, and yield in the organic vetch and conventional wheat systems. Fertilizer scheme affected the magnitude of growing season N_2_O-N loss in the organic vetch system (organic N > fertilizer N-credit) and the timing of loss (organic N delayed N_2_O-N loss vs. urea) and NO_3_-N leaching (urea >> organic N) in the conventional wheat system, but had no effect on yield. Cover crop selection and N fertilization techniques can reduce N leaching and greenhouse gas emissions without sacrificing yield, thereby enhancing N conservation in both organic and conventional conservation agriculture systems.

## Introduction

Agricultural soil management is responsible for ~80% of global annual nitrous oxide (N_2_O) emissions (Davidson, 2009) and 74% of United States N_2_O emissions (U.S. EPA, 2015). An estimated 20% of agriculturally applied N accumulates in our water resources causing algal blooms and disrupting aquatic ecosystems, and ammonia (NH_3_) volatilization increases regional acid deposition (Smil, 1999; Galloway et al., 2004; Suddick et al., 2013). These N losses are, in part, a consequence of management practices that do little to lessen or slow the cycling of N, such as N application timing that is poorly synced with crop N uptake, frequent soil disturbance, and soil left bare during the non-growing season (Mohr et al., 1999; Drinkwater and Snapp, 2007; Jan et al., 2011). Additionally, the cost of N fertilizer has substantially increased in the last decade (USDA-ERS, 2013); thus, off-farm losses are environmentally and economically detrimental, as losses negatively affect farmers' profit.

Conservation agriculture is a management approach employed to decrease N loss in both organic and conventional systems. This approach promotes reduced tillage or no-tillage, use of living or non-living organic residues on the soil surface, and crop rotation and/or intercropping (Scopel et al., 2013). On farm soil resources are preserved, as reduced tillage and residue coverage decrease erosion, and living residues take up residual nutrients from the previous crop. According to the FAO, between 1990 and 2000, conservation agriculture was primarily practiced in North and South America, but between 2001 and 2011, the practice spread across Europe, Asia, Australia, New Zealand, and parts of Africa (FAOSTAT)^1^. Due to the increasing percentage of land area employing these management techniques, it is critical to improve understanding of how conservation agriculture practices influence N loss pathways and how those differences in loss pathways affect yield (Wittwer et al., 2017).

Cover crops, also referred to as living residues, are grown in the off-season, to prevent N export via leaching and/or runoff or to provide N to the following crop via N fixation (McCracken et al., 1994; Drinkwater and Snapp, 2007; Zhou et al., 2012; Finney et al., 2017). Diverse species of cover crops are utilized, depending on the desired ecosystem services and specific environmental/system conditions. Leguminous species are valued for potential N fixation and their contribution to reduced input costs for the following summer crop (Roberts et al., 1998). These cover crops are able to provide N for the following crop and reduce required fertilizer N, but further research is needed in order to understand how reliable N release from cover crop sources is in comparison to fertilizer N as N release from cover crop residues is likely controlled by parameters that vary regionally, seasonally, and with management strategies (Kaye and Quemada, 2017).

For N capture and retention, cereal grass cover crops are recommended because they establish more quickly than legumes, they do not fix additional atmospheric N, and their root growth remains active in cooler temperatures which renders them less prone to winter leaching losses (McCracken et al., 1994; Ranells and Wagger, 1997). Conventional systems may primarily benefit from cereal grasses, as these systems are more likely to contain residual N in the soil profile, while organic farms tend to be more N-limited (Seufert et al., 2012). From an organic matter building perspective, high biomass production is desired by both systems, but organic systems also value biomass for its contribution to weed control during the subsequent growing season. In order to achieve N contribution, N retention, organic matter, and weed suppression—a suite of simultaneous ecosystem services—growing a bi-culture of legumes and cereal grasses may be a beneficial alternative (Teasdale and Abdul-Baki, 1998; Snapp et al., 2005; Mariotti et al., 2015; Finney et al., 2016, 2017).

In addition to choice of cover crop, N loss pathways are also strongly influenced by type of fertilizer. Fertilizers that provide a C source often stimulate heterotrophic microbial activity and release N for nitrification (Fairchild et al., 1999; Mitchell et al., 2013) and those that hydrolyze rapidly prior to assimilating into the soil profile are prone to volatilization (Rochette et al., 2009). Fertilizer N source, along with environmental factors and application technique, dictates N available for plant uptake (Venterea et al., 2011). Ultimately, fertilizer management strategies interact with the presence or absence of organic residues and the tillage regime, and all directly influence environmental factors that control biotic and abiotic N transformations in the soil environment, making it difficult to isolate which combination of practices (i.e., fertilizer N vs. cover crop N) is best for reducing N loss while simultaneously able to achieve competitive yields (Liu et al., 2002; Dyer et al., 2012; Hu et al., 2013; Das et al., 2015). Additionally, N dynamics are known to be spatially and temporally sensitive and thus recommendations must be based on regional or even site–specific data.

In this study, our objective was to investigate the effects of cover crop species and fertilizer schemes on N availability, N loss, and yield in organically and conventionally managed corn systems during a fall/winter cover crop growing season and the following spring/summer corn growing season. Due to the fundamentally different management practices of organic and conventional systems, system-specific N conserving strategies must be developed. Specifically, our objectives were to quantify: (a) the effects of cover crop species (winter wheat vs. hairy vetch vs. a bi-culture of the two species) in an unfertilized, organic system; (b) the effects of fertilizer scheme in an organic system with a hairy vetch cover crop (0 N vs. organic fertilizer vs. a fertilizer N-credit approach that reduces the applied fertilizer by calculating and accounting for the N contribution of the cover crop immediately prior to termination); and (c) the effects of fertilizer type in a conventional corn system planted with a winter wheat cover crop (0 N vs. urea with a urease inhibitor vs. a pelletized organic) on N loss via gaseous N_2_O and NH_3_ emissions, NO_3_ leaching, and N availability to the crops. We hypothesized that (1) the bi-culture cover crop would reduce N loss via lesser leaching in comparison to the hairy vetch cover crop and, though winter wheat would likely have the least N loss, it would reduce available N to the following corn crop and decrease yield; (2) that the fertilizer N-credit approach would enhance N conservation by achieving yields equivalent to a full organic fertilizer application while using less fertilizer N and reduce field N loss as we expected that vetch residue would mineralize more slowly and synchronously with plant uptake compared to fertilizer; and (3) that organic fertilizer in the conventional system would reduce N loss by releasing continuously throughout the season.

## Materials and methods

### Research site

This experiment was established in October 2013 at the University of Kentucky's Horticulture Research Farm in Fayette County, Kentucky in an organic field (37°58′25″N, 84°32′9″W) and a conventional field (37°58′28″N, 84°32′10″W) that were ~150 m apart. The organic field has been under USDA National Organic Program certification since 2009. It was kept fallow and tilled once per year for the three years prior to plot establishment. The conventional field was similarly managed, but was planted with a fall strawberry crop one year prior to plot establishment. Both field sites had been in production for the previous 35 years under conventional tillage regimes. The soil series is a Maury silt loam (fine, mixed, active, mesic Typic Paleudalfs, Table S1). The climate of the region is warm, moist, and temperate with a mean annual temperature of 13.1°C and precipitation of 114.7 cm (NOAA, 2015). However, the year of the study was cooler (mean temperature of 11.5°C) and wetter (143.7 cm precipitation) than the historic average (Kentucky Mesonet)^2^. Specifically, temperatures were cooler than average in January through March and in July, and precipitation was notably higher in December, April, and August.

### Experimental design

In September 2013, fields were spaded with a rotary spader (Imants BV, Reusel, Netherlands). In October 2013, 24 plots (each 5 m by 5 m) were established: 15 in the organic (representing five treatments, three replicates of each) and nine in the conventional field (three treatments, three replicates of each). Treatments in both fields were assigned in a completely randomized design. Winter cover crop treatments were broadcast planted the first week of October and terminated 20 May 2014 in all plots. On 28 May 2014 five rows of a summer corn (*Zea mays indenata*) crop were planted by hand to simulate a no-till planter (91.44 cm between row spacing, 15.24 cm within row spacing) in each plot. Fertilizer N was broadcast applied to selected treatments the same day as corn planting. The corn crop was hand harvested on 6 October 2014.

#### Organic field

In the organic field, five treatments were designed to compare: (1) the effect of cover crop species on N loss and (2) the effect of fertilizer scheme on N loss within systems using a hairy vetch cover crop (Table 1, Figure S1). For the first comparison, three cover crop species were planted: hairy vetch (*Vicia villosa*) seeded at 33.6 kg ha^−1^, winter wheat (*Triticum aestivum*) seeded at 134.5 kg ha^−1^, and a mix (“bi-culture”) of hairy vetch and winter wheat seeded at 22.4 and 67.3 kg ha^−1^, respectively. To capture the effect of cover crop alone, each of the three cover crop treatments were compared under no fertilizer conditions.

**Table 1:** Source and quantity of N input into each of the three system comparisons via cover crops and fertilizer applications.

**System**	**Comparison**	**Cover crop species**	**Cover crop N (kg N ha^−1^)**	**Fertilizer N (kg N ha^−1^)**	**Total N input (kg N ha^−1^)**
Unfertilized organic	Cover crop species	Wheat	27	0	27
		Hairy vetch	78	0	78
		Bi-culture	100	0	100
Fertilized organic	Fertilizer vs. Fertilizer+N-Credit	Hairy vetch	78	0	78
		Hairy vetch	74	168	242
		Hairy vetch	74 + 102	56	232
Fertilized conventional	Urea vs. Organic fertilizer	Wheat	70	0	70
		Wheat	70	168	238
		Wheat	70	168	238

To quantify the effect of fertilizer scheme, two organic N fertilizer treatments applied to a hairy vetch cover crop treatment were compared to a hairy vetch cover crop with no fertilizer added (the same hairy vetch plots used in the cover crop species comparison above). At the time of cover crop termination, hairy vetch biomass was sampled from all plots and was found to contain 70–80 kg N ha^−1^. For the unfertilized treatment, the vetch cover crop N was the only source of N added (averaging 78 kg N ha^−1^). For the organic N fertilizer treatment, 168 kg N ha^−1^ of blended, pelletized, animal byproduct fertilizer (NatureSafe 13-0-0, Griffin Industries LLC, Cold Spring, KY) was added, giving a total of 242 kg N ha^−1^ (74 kg N ha^−1^ from the vetch+168 from the NatureSafe). Due to the atypically cold winter, hairy vetch biomass was low at the site (1,741 kg ha^−1^ dry wt); therefore, for the fertilizer N-credit treatment, additional vetch biomass from an adjacent field was added to these plots to better mimic a more typical year (Smith et al., 1987; Cline and Silvernail, 2002). Vetch biomass sampling after this addition indicated there was 176 kg N ha^−1^ present. To bring the level of added N in the fertilizer N-credit treatment in line with that of the organic N treatment (Sarrantonio, 2012), an additional 56 kg N ha^−1^ of the same organic fertilizer was added. Therefore, the fertilizer N-credit treatment received 232 kg N ha^−1^ in total (Table 1).

The cover crops were terminated via flail mowing on 20 May 2014 (when hairy vetch was at 50% flowering). The corn variety used was 71T77cnv from BlueRiver Organics (114 day, untreated conventional, non-GMO). Between row weed pressure was managed as needed with four mowing events using a BCS Model 853 walk-behind tractor (BCS America, LLC, Portland, OR) with a flail-mower attachment on 13 and 25 June, 10 July, and 22 August 2014. Within-row weed pressure was managed with a single hand cultivation event on 2 July 2014. No weed biomass was removed from the plots. Insect pests were managed with three applications of *Bacillus thuringiensis kurstaki* (Javelin®, Certis, USA, Columbia, MD) and spinosad (Entrust®, Dow Agrisciences, Indianapolis, IN) using a spreader sticker (Nu-film 17, Miller Chemical and Fertilizer Corporation, Hanover, PA) (Table S2).

#### Conventional field

In the conventional field, three treatments were designed to compare the effect of two different fertilizers on N loss in conventional systems with a winter wheat (*T. aestivum*) cover crop seeded at 134.5 kg ha^−1^ (Table 1, Figure S1). The N treatments were 0 N, 168 kg ha^−1^ N applied as urea 40-0-0 with a urease inhibitor (AgrotainUltra®), and 168 kg ha^−1^ N applied as the same fertilizer used in the organic treatments. Animal derived N sources such as manure are often used as an alternative to ammonium nitrate or urea, but in order to eliminate adding additional phosphorous and/or potassium a 13-0-0 packaged source of organic fertilizer was used in this study (NatureSafe 13-0-0, Griffin Industries LLC, Cold Spring, KY). The cover crop was terminated via flail mowing followed by glyphosate application (Roundup Pro; Monsanto, St. Louis, MO, U.S.A.) on 20 May 2014. Corn was planted and fertilizer applied on 28 May 2014. The corn variety used in the conventional field was REV24BHR93 from Terral Seed, Inc. Weeds were managed with one additional application of glyphosate on 10 July 2014 and no insect pest management was deemed necessary upon weekly scouting (Table S2).

### Measured parameters

#### Gaseous emissions

The static chamber method was employed to measure gaseous emissions (Parkin and Venterea, 2010). In each of the 24 plots (8 treatments, 3 replicates), a rectangular stainless steel anchor (16.35 × 52.70 × 15.24 cm) was inserted into the soil so that the top was nearly flush with the soil surface. Anchors were inserted at random locations in each plot 10 days after cover crop seed was broadcast in the fall. Anchors were removed prior to cover crop termination in order to avoid damage from heavy machinery. The day of corn planting, the anchors were re-inserted into the plots and were placed perpendicular to the corn rows so that they were between two corn plants and spanning soil surface area both within and between rows.

To measure nitrous oxide (N_2_O) and ammonia (NH_3_), a “cap” made from an identical stainless steel chamber, equipped with a vent tube and lined with Teflon© tape (Bytac©, Saint Gobain Performance Plastics, Paris, France) was attached to the anchor to create a sealed chamber. The chamber was connected to a photoacoustic spectroscopy gas analyzer (Innova Air Tech Instruments Model 1412, Ballerup, Denmark) via Teflon© tubing. Measurements were taken continuously for 10 min on the days of sampling and NH_3_ and N_2_O concentrations (mg L^−1^) were recorded simultaneously. Gaseous flux was calculated using the equations described by Iqbal et al. (2013). Annual fluxes were estimated by interpolating linearly between sampling dates, calculating the area under the curve using the trapezoidal rule. During sampling periods, additional environmental parameters were also recorded, including soil moisture at 5 cm depth (DELTA-T HH2 moisture meter using a ML2x 6 cm theta probe, Delta-T Devices, Cambridge, England), soil temperature at 5 cm depth, and ambient air temperature (Taylor Digital Pocket Thermometer, Model 9878E, Taylor Precision Products, Oak Brook, IL).

Measurements commenced on 28 October 2013 and continued until 29 October 2014. Sampling intensity varied throughout the experiment, with increased intensity during periods when fluxes were expected to be affected by management practices. During the cover crop growing season (28 October 2013–20 May 2014) measurements were taken twice a month (cover crop growth period). Following corn planting and fertilization (28 May 2014), measurements were taken daily for a week and then every other day until 12 June 2014, on which date fluxes from fertilized treatments appeared similar to those from unfertilized treatments (post fertilization intensive period). During the rest of the corn-growing season, measurements were taken every seven to 10 days, returning to a twice a month sampling scheme in October (post fertilization period). Measurements were taken between 10:00 a.m. and 3:00 p.m.

#### Nitrogen leachate

Ion exchange resin lysimeters were used to measure NO_3_-N and NH_4_-N leachate, after Susfalk and Johnson (2002). Cation and anion exchange resins (25 g) (LANXESS NM-60, Klenzoid Equipment Company, Wayne, PA) were placed between Nitex® nylon cloth and sand layers that were enclosed in polyvinyl chloride tubes 5 cm in diameter. Lysimeters were deployed during cover crop growth from 10 October 2013 until 16 May 2014 and during corn growth from 16 May 2014 until 20 October 2014. Two lysimeters were installed at 40 cm depth in each plot under an undisturbed soil profile, ensuring good contact with the soil. The first round was placed randomly in each plot, but during the corn growing season, one was placed within and one between the corn rows.

When resin lysimeters were collected, inorganic ammonium and nitrate was extracted by shaking the resin in 100 ml of 2 M KCl for 1 h. Ammonium concentration was determined using a modification of the Berthelot reaction (Chaney and Marbach, 1962) and nitrate quantified via reduction to nitrite using a copperized cadmium reduction microplate device (ParaTechs Co., Lexington, KY) as described by Crutchfield and Grove (2011). Colorimetric analysis was conducted using a microplate reader (Molecular Devices, VERSAmax, Sunnyvale, CA).

#### Soil inorganic nitrogen

To monitor soil N dynamics, cation and anion exchange resin bags were made with 10 g of resin (LANXESS NM-60, Klenzoid Equipment Company, Wayne, PA) tied in a porous material (Gibson et al., 1985). Each month from November 2013 to November 2014, three resin bags per plot were inserted at 15 cm depth and those from the prior month were removed for analysis of inorganic ammonium and nitrate. There were two instances (January to mid-February and mid-February to early April) when the resin bags were deployed for 6 weeks as opposed to 1 month, as the ground was frozen and it was not possible to remove the resin bags. After removal from the field, resin bags were rinsed with deionized water and extracted with 40 mL 2.0 M KCl. Extract was stored overnight at 4°C and analyzed colorimetrically as described above.

#### Plant biomass sampling

Beginning in May 2014, cover crop biomass residue was sampled monthly through September. The first sampling was conducted the day of cover crop termination. During the months of June through September, the decomposing cover crop residue on the soil surface was collected. Two samples were collected randomly from each plot (25 × 25 cm quadrat), were dried (55°C for 48 h), and then weighed. The same location was never sampled twice. Samples were processed on a grinding mill to pass through a 1 mm sieve (Cyclotec 1093, FOSS™, Eden Prairie, MN) and sub-samples were then ground on a ball grinder (Cianflone Scientific Instrument Corporation, Pittsburgh, PA) or a jar-mill (U.S. Stoneware, East Palestine, OH). Cover crop biomass was analyzed for C and N content via flame combustion (Flash EA 1112 elemental analyzer, CE Elantech Inc., Lakewood, CA).

Corn was harvested by hand on 6 October 2014. Yield was calculated from the grain produced by the inner three rows of each plot. Corn was dried at 55°C for 48 h and then allowed to air-dry for 5 weeks. Corn was shelled, weighed, and a grain sub-sample was collected and dried (55°C for 24 h) to correct for moisture content.

### Statistical analyses

This experiment was designed to investigate the effects of cover crop species and fertilizer scheme on N loss in organically and conventionally managed corn systems. Given the substantial historical and current management differences between the organic and conventional systems, these fields were analyzed separately. From eight total treatments, three separate contrasts were developed that addressed the three specific objectives of the study. Within the organic field, unfertilized plots differing in cover crop species were compared (hairy vetch vs. wheat vs. bi-culture), and treatments planted with hairy vetch, but receiving different fertilizer schemes (0 N vs. fertilizer N-credit vs. organic N), were compared. In the conventional field, the effect of fertilizer type was compared across treatments (0 N vs. organic N vs. urea). General and mixed linear models (proc GLM, proc mixed) (9.3 SAS Institute Inc., Cary, NC) were utilized for the analyses. A repeated measures ANOVA model was employed for parameters measured multiple times over the study year. When significant main effects were identified, a least squares means statement was used to produce pairwise comparisons across treatments on individual measurement dates.

## Results and discussion

### Organic field: cover crop comparisons

#### Cover crop growing season

**Figure 1:**
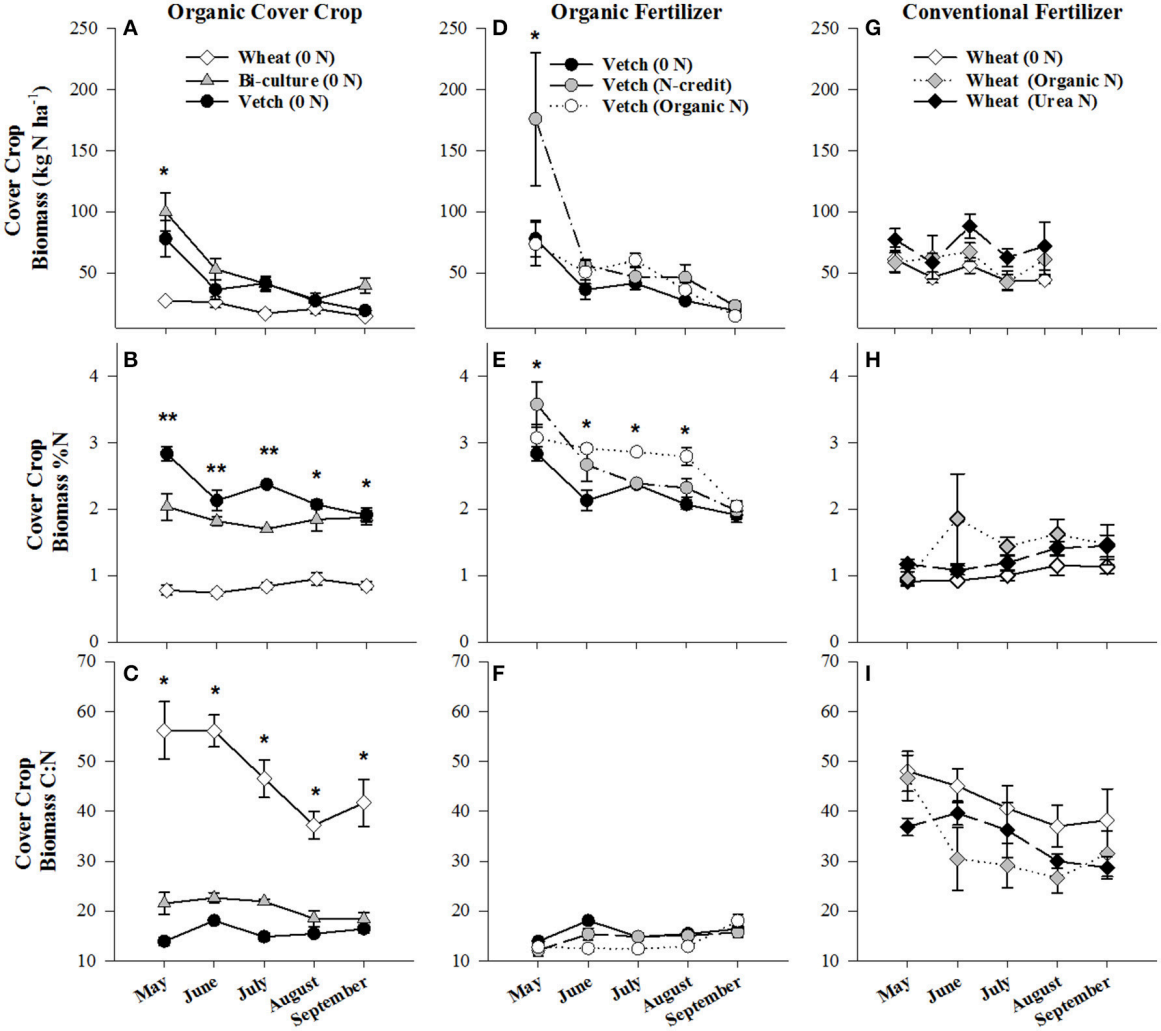
Average (±SE) aboveground cover crop biomass residue N content (kg N ha^−1^), %N, and C:N ratio harvested from the soil surface from May to September 2014 in the organic cover crop treatments **(A–C)**, the organic N-fertilizer treatments **(D–F)**, and the conventional N-fertilizer treatments **(G–I)**. Asterisks indicate points in time where significant effects between treatments were observed and double asterisks indicated points in time where all treatments significantly differed from one another. Cover crop species are indicated in the legends, where 0 *N* = no applied fertilizer, fertilizer N-credit = 56 kg ha^−1^ organic fertilizer+additional hairy vetch, Organic *N* = 168 kg ha^−1^ organic fertilizer, Urea *N* = 168 kg ha^−1^ urea+urease inhibitor.

Biomass of the cover crops at termination was significantly greater in the vetch-wheat bi-culture and wheat plots than the hairy vetch alone (*p* < 0.0096, *p* < 0.0118, respectively), with dry weights of 4,968 (bi-culture), 3,590 (wheat), and 2,791 (hairy vetch) kg ha^−1^. Nitrogen content of biomass followed similar trends and was 100 kg N ha^−1^ in the bi-culture, 78 kg N ha^−1^ in hairy vetch alone, and 27 kg N ha^−1^ in the wheat (Figure 1A). During cover crop growth (October–May), soil moisture varied from a low of 13% on 28 October 2013 to a high of 35% on 18 February 2014, but was comparable across treatments, differing slightly (<6%) at only three time points (Figure S2). Similarly, overall soil temperature did not vary substantially across treatments (Figure S3).

**Figure 2:**
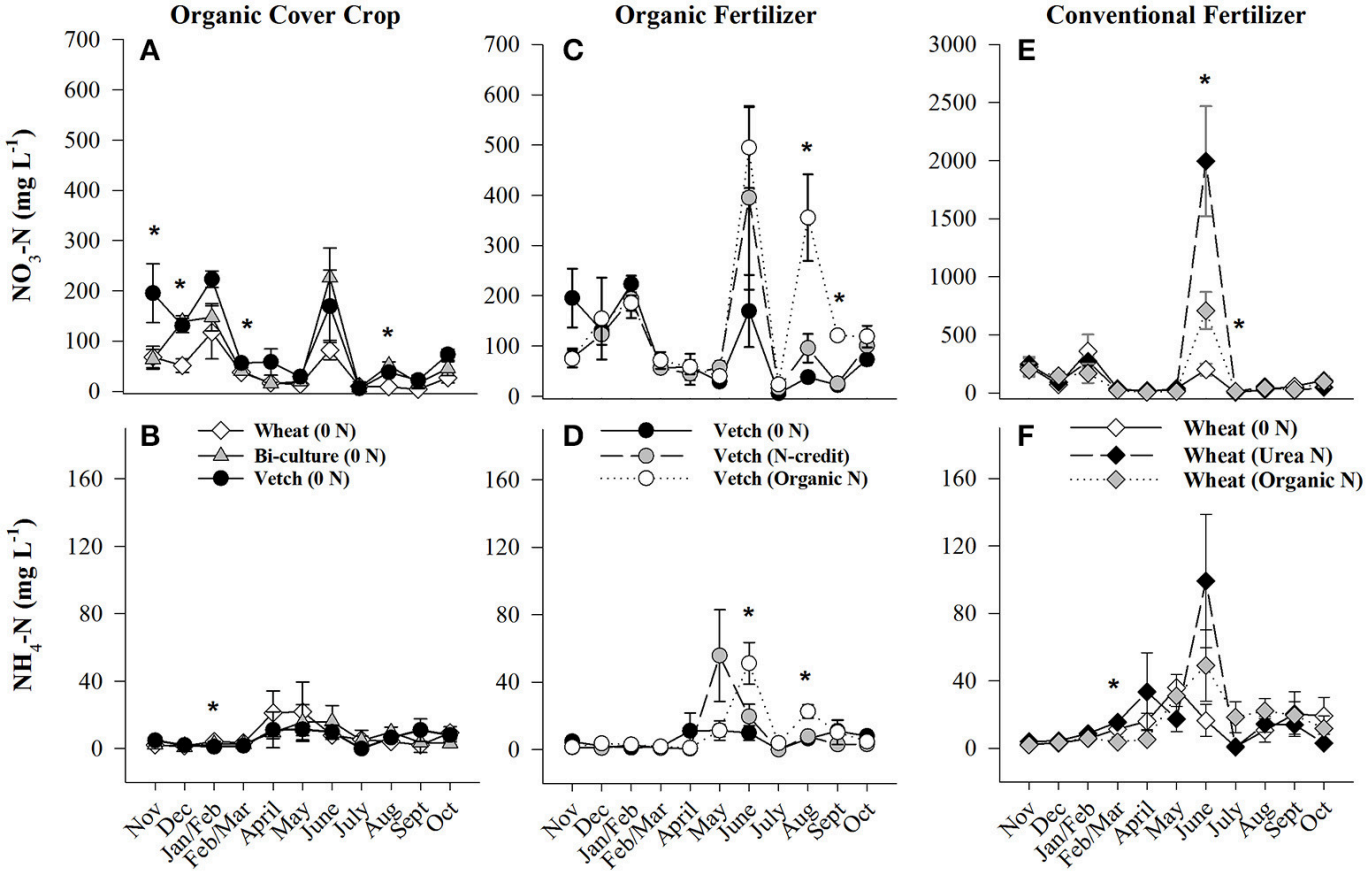
Average (±SE) NO_3_-N and NH_4_-N concentrations extracted from soil resin bags (15 cm depth) from November 2013 until October 2014 in the organic cover crop treatments **(A,B)**, the organic N-fertilizer treatments **(C,D)**, and the conventional N-fertilizer treatments **(E,F)**. Asterisks indicate points in time where significant effects between treatments were observed. Note that scales differ across graphs (**A,C** vs. **E**). Cover crops were terminated and fertilizers were applied on 20 and 28 May 2014, respectively. Cover crop species are indicated in the legends, where 0 *N* = no applied fertilizer, Organic *N* = 168 kg ha^−1^ organic fertilizer, fertilizer N-credit = 56 kg ha^−1^ organic fertilizer+additional hairy vetch, Urea *N* = 168 kg ha^−1^ urea+urease inhibitor.

**Figure 3:**
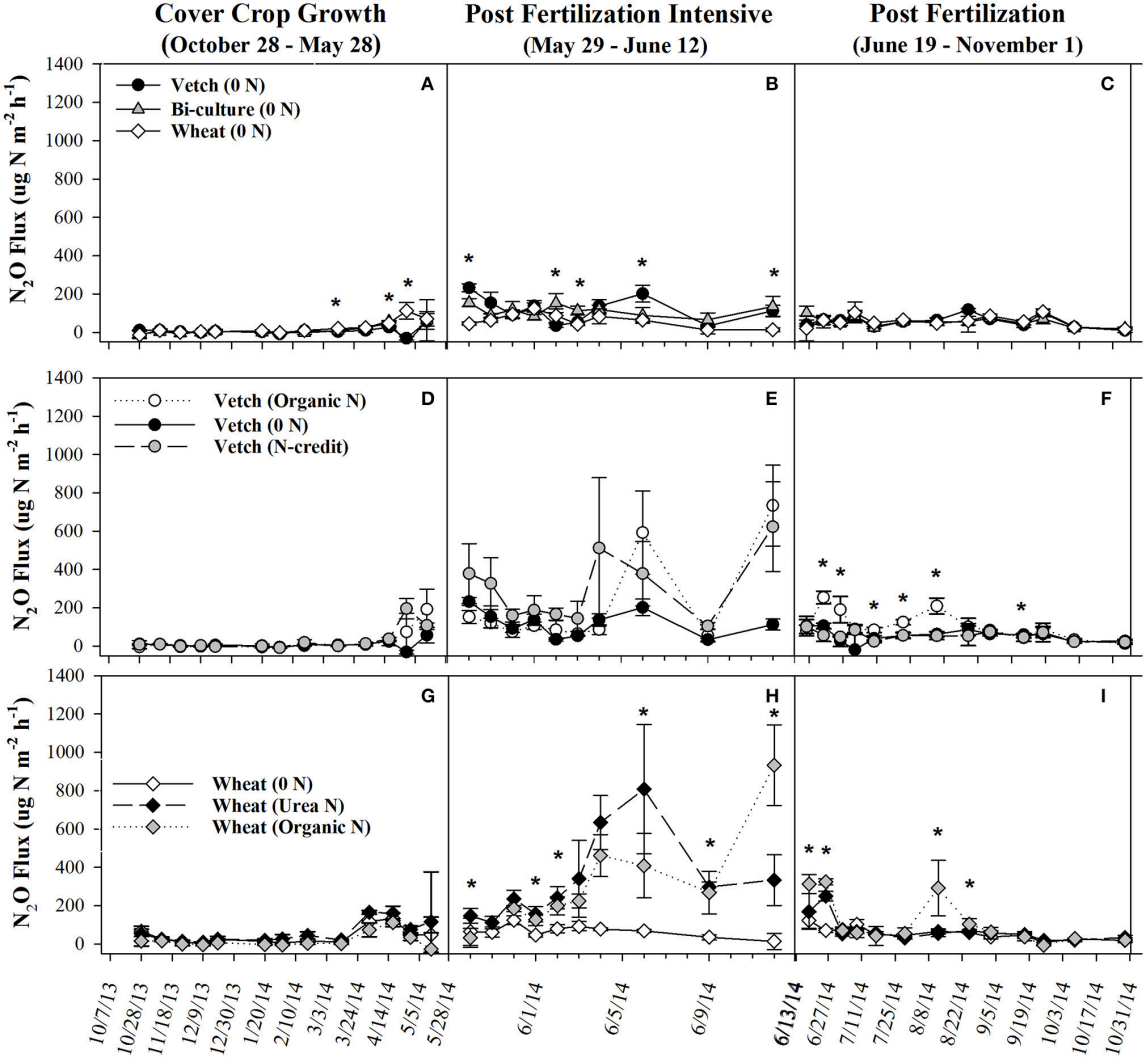
Average (±SE) N_2_O flux for organic cover crop treatments **(A–C)**, organic N-fertilizer treatments **(D–F)**, and conventional N-fertilizer treatments **(G–I)** across three different time periods (twice a month during the cover crop growth period, daily to every other day after fertilizer was applied and, after fluxes from fertilized treatments appear similar to unfertilized treatments, every 7–10 days during corn growth) from October 2013 to November 2014. Asterisks indicate points in time where significant effects between treatments were observed during the measurement period. Cover crop species are indicated in the legends, where 0 *N* = no applied fertilizer, fertilizer N-credit = 56 kg ha^−1^ organic fertilizer+additional hairy vetch Organic *N* = 168 kg ha^−1^ organic fertilizer, Urea *N* = 168 kg ha^−1^ urea+urease inhibitor.

Soil resin NO_3_-N concentrations were highest in the hairy vetch treatment prior to cover crop termination in January/February, but NH_4_-N concentrations were similar across treatments (Figures 2A,B). Nitrogen loss measured as NO_3_-N leachate differed between cover crop species, with hairy vetch > bi-culture > wheat (Table 2). Though N_2_O-N fluxes were slightly higher in the wheat treatment on 3 March and 17 and 29 April 2014 (Figure 3A), linear contrasts indicated there were no significant differences in either N_2_O-N or NH_3_-N loss across treatments when analyzed across the entire cover crop growing season (Table 2).

**Table 2:** Average NO_3_-N leachate and cumulative gaseous N_2_O-N and NH_3_-N fluxes pre-fertilization (Oct–May) and post-fertilization (May–Oct), the percentage of fertilizer N diverted into gaseous N loss pathways, and average corn yield.

**Treatment**	**Fertilizer N (kg N ha^−1^)**	**NO_3_-N Leachate (Oct–May) (mg L^−1^)**	**NO_3_-N Leachate (May–Oct) (mg L^−1^)**	**Cumulative N_2_O-N (Oct–May) (kg N ha^−1^)**	**Cumulative N_2_O-N (May–Oct) (kg N ha^−1^)**	**Cumulative NH_3_-N (Oct–May) (kg N ha^−1^)**	**Cumulative NH_3_-N (May–Oct) (kg N ha^−1^)**	**Apparent Fertilizer lost as % NH_3_-N^†^**	**Apparent Fertilizer lost as % N_2_O-N^†^**	**Corn Yield (Mg ha^−1^)**
**ORGANIC COVER CROP COMPARISONS**										
Hairy vetch	0	443^a^	217^a^	0.79^a^	1.79^a^	−4.01^a^	8.71^a^	–	–	4.30^a^
Wheat	0	222^c^	19^b^	1.16^a^	2.13^a^	−4.40^a^	7.27^a^	–	–	1.46^b^
Bi-culture	0	249^b^	106^a^	0.65^a^	2.05^a^	28.09^a^	18.91^a^	–	–	4.92^a^
**ORGANIC HAIRY VETCH FERTILIZER COMPARISONS**										
0 N	0	443^a^	217^a^	0.79^a^	1.79^b^	−4.04^a^	8.71^c^	–	–	4.30^b^
N-Credit	154	468^a^	249^a^	1.99^a^	3.10^b^	22.75^a^	12.81^b^	2.66	0.63	8.85^a^
Organic N	164	304^a^	307^a^	1.31^a^	4.47^a^	7.62^a^	39.09^a^	18.52	1.43	9.91^a^
**CONVENTIONAL WHEAT FERTILIZER COMPARISONS**										
0 N	0	263^a^	63^b^	1.97^a^	1.83^a^	13.69^b^	−5.80^b^	–	–	6.66^b^
Urea N	168	227^a^	602^a^	3.04^a^	3.54^a^	36.95^a^	24.29^ab^	17.92	1.02	16.87^a^
Organic N	168	154^a^	109^b^	0.86^a^	5.05^a^	−8.10^c^	43.07^a^	29.09	1.92	12.62^ab^

*Different letters indicate significant difference (p < 0.05)*.

During cover crop growth, low soil temperatures and N availability likely contributed to low gaseous loss for all treatments (Parsons et al., 1991). Thus, the primary N loss pathway during this time was NO_3_-N leaching. Our result of hairy vetch having the greatest NO_3_-N loss via leachate is consistent with other work that has evaluated legume vs. grass cover crop effects on N leaching (McCracken et al., 1994; Ranells and Wagger, 1997). The low biomass produced by the hairy vetch treatment (only 56–78% of the biomass of the other treatments) and the release of fixed N to the soil through rhizodeposition (Fustec et al., 2011) likely contributed to greater leaching. The fact that the bi-culture had the greatest biomass accumulation, highest N content, and lower NO_3_-N leachate than the hairy vetch treatment is consistent with other studies that have shown when hairy vetch is grown in bi-culture with wheat, biomass production and the winter hardiness of the legume is increased (Teasdale and Abdul-Baki, 1998; Snapp et al., 2005). For organic production, higher cover crop biomass and N content in the bi-culture may provide a slow-release source of N for the subsequent corn crop.

#### Corn growing season

**Figure 4:**
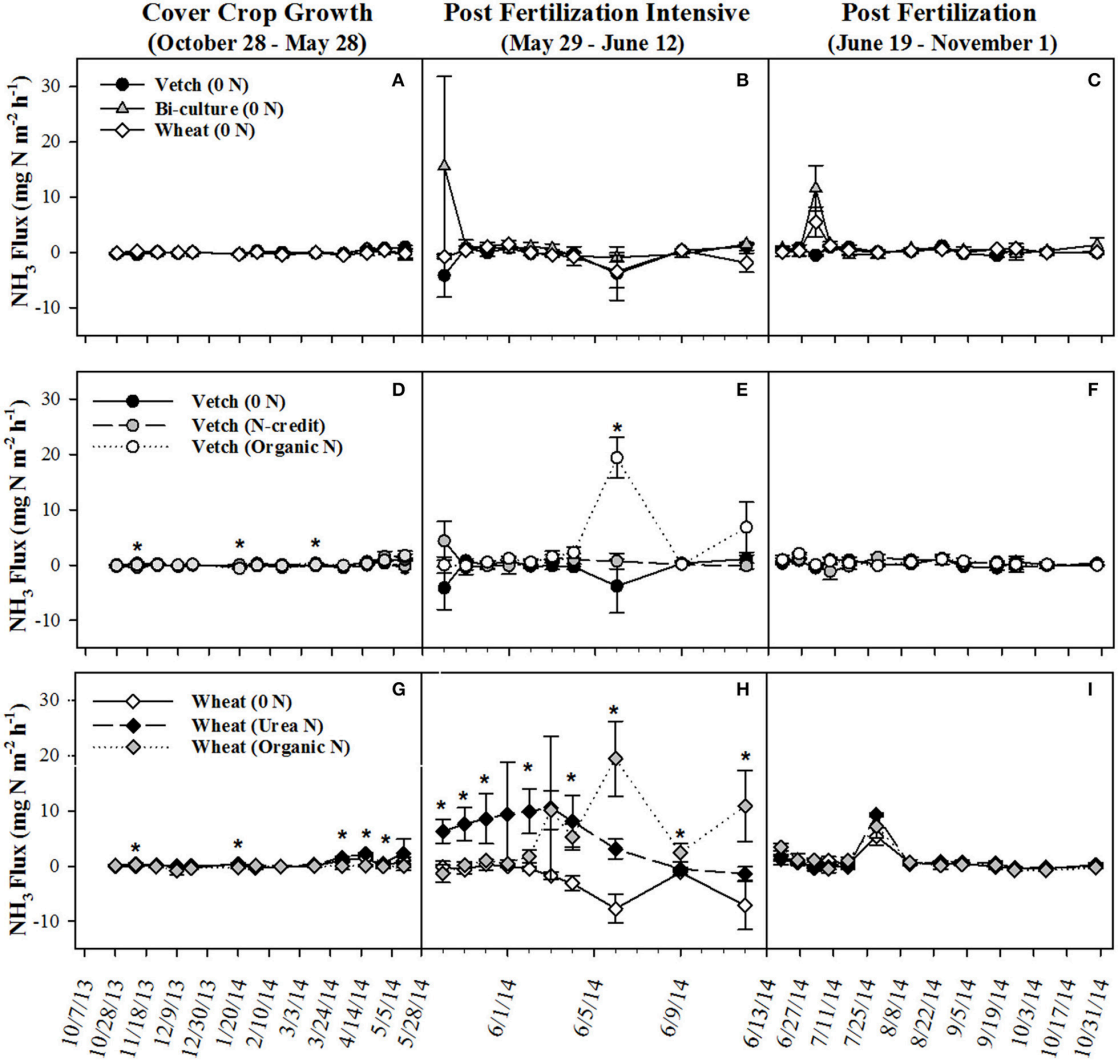
Average (±SE) NH_3_ flux for cover crop treatments **(A–C)**, organic N-fertilizer treatments **(D–F)**, and conventional N-fertilizer treatments **(G–I)** across three different time periods (twice a month during the cover crop growth period, daily to every other day after fertilizer was applied and, after fluxes from fertilized treatments appear similar to unfertilized treatments, every 7–10 days during corn growth) from October 2013 to November 2014. Asterisks indicate points in time where significant effects between treatments were observed during the measurement period. Cover crop species are indicated in the legends, where 0 *N* = no applied fertilizer, fertilizer N-credit = 56 kg ha^−1^ organic fertilizer+additional hairy vetch Organic *N* = 168 kg ha^−1^ organic fertilizer, Urea *N* = 168 kg ha^−1^ urea+urease inhibitor.

Post-termination of the cover crops (May–October), N content and %N of hairy vetch and bi-culture residue declined, indicating N mineralization during decomposition, whereas the N content of the wheat residue stayed fairly constant at a low level and the %N slightly increased, indicating N immobilization (Figures 1A,B). These trends reflected significant differences in residue C:N (wheat > bi-culture > vetch; Figure 1C), and likely contributed to higher NO_3_-N levels measured in August soil resin bags (Figure 2A), higher NO_3_-N leachate values (Table 2), higher N_2_O-N emissions during the post-fertilization intensive measurement period (Figure 3B), and higher corn yield (Table 2) in hairy vetch and bi-culture treatments vs. wheat. No significant differences between treatments were observed for total NH_3_-N or N_2_O-N emissions (Table 2), for N_2_O-N emissions during the post-fertilization period (Figure 3C), or for NH_3_-N emissions during specific measurement periods (Figures 4A–C).

During the corn growing season, N leachate concentrations were lower than during the cover crop growing season, but N_2_O-N emissions were greater. A combination of increased N availability in the system after cover crop termination, warmer soil temperatures, and adequate soil moisture (Figures S2, S3) were likely causal (Colbourn, 1993; DeKlein and VanLogtestijn, 1996). Similar to the research of Wisal et al. (2011), who found that soils amended with low C:N ratio residues undergo decreased N immobilization and increased N_2_O-N emissions, cover crop C:N appeared to influence gaseous N emissions. N_2_O-N fluxes increased after cover crop termination for the vetch (from 57 to 232 μg N m^−2^ h^−1^) and bi-culture treatments (62–155 μg N m^−2^ h^−1^), but decreased in the wheat system (from 70 to 46 μg N m^−2^ h^−1^), as the high C:N wheat cover crop biomass residue induced N immobilization rather than mineralization.

Although the wheat cover crop had the lowest N loss (primarily due to low N leaching), N immobilization into the residue after termination (Wagger, 1989; Wyland et al., 1995; McSwiney et al., 2010) resulted in significantly lower corn yields. Though capable of reducing N loss, systems cover cropped with wheat would require significant quantities of N fertilizer to obtain the yields necessary for a viable production system, perhaps negating its N loss benefit. The fact that the bi-culture treatment had less NO_3_-N leachate loss during cover crop growth and similar post-termination leaching, gaseous N loss, and corn yield compared to the hairy vetch treatment suggests that legume-grass cover crop mixtures have potential for reducing N loss in organic conservation agricultural systems. Further cover crop management research is essential as all treatments in this system were unfertilized and most likely N limited, as corn yields were half of those produced in the fertilized organic treatments also evaluated in this experiment. With higher fertilizer N applications, the effect of cover crop species on N loss pathways and yield may change (Alvarez et al., 2017). Additionally, longer-term studies are needed as soil properties are affected by the addition of organic residues over time (Saha and Mishra, 2009; Saptoka et al., 2012; Mbuthia et al., 2015).

### Organic field: fertilizer scheme comparisons

#### Cover crop growing season

As all treatments were planted with hairy vetch and fertilizers had not yet been applied, measured soil resin N concentrations, N leachate, and gaseous N_2_O-N loss parameters did not differ during the cover crop growing season (Figures 2C,D, 3D, Table 2). Though there were slight differences in NH_3_-N emissions on three dates (Figure 4D), treatments were not significantly different across the entire measurement period (Table 2) as gaseous N losses were low at this time as, most likely limited by soil temperatures (Figure S3; Parsons et al., 1991; Colbourn, 1993; DeKlein and VanLogtestijn, 1996). N leaching was the dominant N loss pathway.

Due to the unusually low winter temperatures experienced at the site, hairy vetch biomass production was low overall (Figure 1D). To simulate a more representative year, supplemental vetch biomass (~2,000 kg ha^−1^) was harvested from an adjacent organic field and added as residue to the fertilizer N-credit treatment. This resulted in hairy vetch biomass and N content being significantly greater in the fertilizer N-credit treatment than the unfertilized or organic treatments at cover crop termination (Figure 1D). Had this biomass addition not occurred, the fertilizer N-credit approach would have been nearly identical to the organic (fertilizer alone) treatment, and not representative of typical vetch production at the site, thus limiting our understanding of the N dynamics of a conservation agriculture system predominantly fertilized with vetch biomass.

#### Corn growing season

Although initial cover crop N content was greatest in the fertilizer N-credit treatment, by June, there was no difference between treatments (Figure 1D), as the hairy vetch residue underwent rapid decomposition (Hadas et al., 2002; Acosta et al., 2011). N content and %N of residue declined similarly in all treatments during decomposition (Figures 1D,E,F). Post-fertilization, soil NO_3_-N and NH_4_-N dynamics differed between treatments. Overall, soil resin NO_3_-N and NH_4_-N tended to be greatest in the organic N fertilized treatment, followed by the fertilizer N-credit treatment, and lowest in the unfertilized treatment (Figures 2C,D). Despite observed treatment differences in post-fertilization soil resin extracted N, there were no differences in NO_3_-N or NH_4_-N leachate values between treatments (Table 2). However, there were differences in gaseous N emissions.

During the corn growing season, N gaseous loss pathways were influenced by the quantity and form (vetch biomass vs. organic fertilizer) of applied N. Overall, the organic N treatment had the highest N_2_O-N emissions (Table 2), but the fertilizer N-credit treatment had higher initial emissions (Figure 3E). A greater quantity of cover crop residue in the fertilizer N-credit treatment led to soil moisture being consistently greater (by 2–6%) during the post-fertilization intensive measurement period (29 May−12 Jun) vs. the unfertilized or organic fertilized treatments (Figure S2), most likely via prevention of evaporative loss from the soil surface. These conditions may have stimulated microbial activity, N mineralization, and subsequent, denitrification and N_2_O fluxes (Colbourn, 1993; Schomberg et al., 1994; DeKlein and VanLogtestijn, 1996; Dobbie et al., 1999; Hu et al., 2013). Periods of high soil moisture continued to induce denitrification throughout the growing season; N_2_O-N gas flux peaks in all treatments were often linked with rain events, particularly in the organic N treatment. Following a rain event on 4 June 2014, emissions increased dramatically in the organic N treatment and then remained greater than emissions from the fertilizer N-credit and unfertilized treatments for the remainder of the corn growing season (19 June−31 October; Figure 3F).

N in slow release, organic fertilizers, such as that applied in this study, commonly undergo mineralization followed by denitrification after rain events (Sistani et al., 2011; Venterea et al., 2011; Halvorson and Del Grosso, 2012, 2013). While maintaining a source of available N over the course of the growing season is ideal for plant growth, temporal asynchrony between microbial activity and plant N uptake, such as can occur during wet-dry cycles, can result in significant N_2_O-N loss (Augustine and McNaughton, 2004; Schwinning and Sala, 2004; Dijkstra et al., 2012; Parkin and Hatfield, 2014).

NH_3_-N emissions were also greatest in the organic fertilizer treatment, especially during the post-fertilization intensive measurement period, with significant fluxes occurring on 6 June 2014 (Figures 4E,F), following a rain event. Similar to rainfall effects on N_2_O-N fluxes, precipitation most likely induced fertilizer mineralization, increasing soil NH_3_ concentrations and, as soil moisture declined, volatilization occurred (Rochette et al., 2009). By subtracting the unfertilized gaseous N emissions calculated over the corn growing season from the fertilized treatments and dividing by the total amount of N applied in the fertilized treatments, the percentage of the applied N that was diverted into N_2_O-N or NH_3_-N loss pathways was estimated (Parkin and Kaspar, 2006). This approach estimated that 1.4% of the applied N in the organic N treatment was lost as N_2_O-N, whereas only 0.6% of applied N was lost as N_2_O-N in the fertilizer N-credit treatment (Table 2). For gaseous NH_3_-N emissions, ~18.5% and <3% of applied N lost as NH_3_-N from the organic N and fertilizer N-credit treatments, respectively (Table 2).

These results suggest that more N was released from the organic fertilizer than the fertilizer N-credit treatment. Yet, corn yield was similar between these two treatments (and was significantly greater than the unfertilized treatment; Table 2). Though more inorganic N was released in the organic fertilizer treatment, it may not always have been accessible for corn plant uptake or, as other studies have shown, at a higher level of N supply the corn in the organic fertilizer treatment may have used N less efficiently (Moll et al., 1982). It appears that greater N availability in the organic fertilizer treatment later in the season stimulated gaseous emission losses rather than plant uptake and yield. This research suggests that increased incorporation of N supplied by a cover crop into fertilization schemes may decrease N loss and promote more efficient N use by improving the synchronicity of N release with corn plant demand compared to organic fertilizer applications.

### Conventional field: fertilizer type comparisons

#### Cover crop growing season

At termination, wheat cover crop biomass averaged 6,410 kg ha^−1^ and contained ~70 kg N ha^−1^. There were no significant differences across treatments in biomass or N content as all were planted with the same cover crop species (Figure 1G). Similarly, soil NO_3_-N and NH_4_-N concentrations did not differ across treatments during this time period, as fertilization had not yet occurred (Figures 2E,F). However, during February/March, soil NH_4_-N was slightly lower in the organic N fertilizer treatment (urea N, unfertilized > organic N; Figure 2F). There was no difference in N loss via leachate or N_2_O-N emissions (Figure 3G), but differences in gaseous NH_3_-N loss did occur (Table 2). On several dates, NH_3_-N emissions were slightly elevated in the urea N and unfertilized treatments (Figure 4G), reflecting slightly higher soil NH_4_-N in these treatments at those times (Figure 2F). However, when pre-fertilization total NH_3_-N emissions were calculated, all three treatments differed from each other (urea N > unfertilized > organic N; Table 2). Soil moisture during this time was, on average, 2% greater in the urea N vs. the organic N treatment (Figure S2), though a means comparisons test failed to identify specific dates where the treatment effect was significant.

Higher soil resin NH_4_-N and gaseous NH_3_-N losses in the urea treatment during this time period are difficult to explain, as all treatments were planted in the same cover crop species (wheat) and had not received any fertilizer. The only difference apparent between treatments at this time was the presence of volunteer big flower vetch (*Vicia grandiflora*): it was most abundant in the urea treatment (average percent cover ~7%), followed by the organic fertilizer (~5%), and the unfertilized (~4%). Perhaps the presence of this weed or other unknown factors contributed to observed treatment differences.

#### Corn growing season

Post-termination of the winter wheat cover crop and post-fertilization of the organic N and urea N treatments (May–October), N dynamics in the decomposing cover crop residue did not significantly differ between treatments, though residue N content varied across the time period; %N steadily increased (indicating immobilization of N) and C:N ratios declined (Figures 1G–I). However, soil N dynamics differed between treatments. In June, soil resin NO_3_-N concentrations were significantly different between all three treatments (urea N > organic N > unfertilized), and in July both fertilized treatments had greater NO_3_-N concentrations than the unfertilized treatment (Figure 2E). On average, NH_4_-N tended to be greater in both fertilized treatments vs. the unfertilized treatment, but means comparisons failed to identify specific months during which soil NH_4_-N significantly differed, finding only that urea N was marginally greater than the unfertilized treatment for the month of June (*p* = 0.0552).

Gaseous N loss and leaching were strongly affected by the quantity and type of fertilizer applied, with gaseous and leaching N losses in the unfertilized treatment being minimal compared to fertilized treatments. The urea N treatment had 5x more NO_3_-N leachate loss than either the organic N or the unfertilized treatments during this time period (Table 2). Some urea hydrolysis likely occurred prior to corn plant germination; thus, N may have leached down into the soil profile preceding corn plant establishment (Quisenberry and Phillips, 1976), which is supported by the high resin NO_3_-N and NH_4_-N concentrations measured in the urea treatment in June (Figure 2E). To reduce NO_3_ leaching loss associated with urea N applications, a split application or application after corn plant establishment might be necessary (Meisinger and Delgado, 2002).

During the post-fertilization intensive measurement period (29 May–12 June), N_2_O-N emissions were greater in both the fertilized treatments vs. the unfertilized (Figure 3H). On 6 June 2014, N_2_O-N emissions were more than 8x greater in the urea N treatment than the unfertilized treatment, and on 12 June 2014 they were more than 9x greater in the organic N treatment than the unfertilized treatment. On several dates during the remainder of the corn growing season (19 June–31 October), N_2_O-N emissions from the organic N treatment remained significantly higher than the other treatments (Figure 3I). However, these date-specific trends were not strong enough to significantly influence the entire post-fertilization N_2_O-N flux estimates (29 May–31 October), as no differences in N_2_O-N emissions between treatments were found (Table 2). Approximately 2% of fertilizer was lost as N_2_O-N in the organic N treatment, whereas only 1% was lost from the urea N (Table 2).

Higher N_2_O-N loss in the organic N treatment was not unexpected as the fertilizer was not only 13% N, but 40% C (Kirk Carls, Nature Safe Natural and Organic Fertilizers, personal communication, 14 March 2015). Numerous studies have found that fertilizers that provide a C source in conjunction with N stimulate microbial activity and, subsequently, N loss (Limmer and Steele, 1982; Hayakawa et al., 2009; Chantigny et al., 2010; Sistani et al., 2011; Mitchell et al., 2013). N_2_O-N emissions from the organic fertilizer treatment in our study were similar to those calculated by Sistani et al. (2011) in a Kentucky no-till corn system applied with poultry litter. The higher N_2_O-N flux measured in the urea treatment on 6 June 2014 may be the result of urea hydrolyzing more readily than the organic fertilizer, enriching the soil with inorganic N prior to the rain event on 4 June (Figures 2E,F; Parkin and Hatfield, 2014). After this date, the slower N release nature of the organic fertilizer caused higher N_2_O-N emissions from this treatment for majority of the remainder of the study.

NH_3_-N emissions were also stimulated by fertilizer additions (Figure 4H). Temporally similar to N_2_O-N trends, the urea N treatment had greater emissions during the first part of the post-fertilization intensive measurement period while the organic N treatment had greater emissions during the latter half of the measurement period. Despite substantial fertilizer effects early on, treatment differences did not persist across the longer-term post-fertilization period (Figure 4I). When summed across the entire post-fertilization period (29 May–31 October), NH_3_-N emissions were significantly greater in the organic N vs. unfertilized treatments (Table 2). Approximately 29% of fertilizer was lost as NH_3_-N in the organic N treatment, whereas only 18% was lost from the urea N (Table 2).

Christianson et al. (2012) reported that up to 30% of N can be volatilized after broadcast urea N applications, and, in systems where organic residue is present, broadcast urea may be particularly vulnerable to volatilization as the residue acts as a barrier between the soil surface and the fertilizer (Mohr et al., 1998; Rochette et al., 2009; San Francisco et al., 2011). The urease inhibitor included in our urea fertilizer treatment was likely responsible for mitigating volatilization to some degree prior to precipitation events that incorporated the fertilizer into the soil profile (a rain event occurred the night following application, ~0.25 cm, and another on 4 June 2014, ~4.72 cm). After contact with the soil, volatilization was likely minimal, as the chemistry at this field site is not considered particularly susceptible to volatilization (Table S1). NH_3_-N volatilization of urea N subsided after the rain event on 4 June 2014, whereas this same event caused a spike in NH_3_-N emissions in the organic N treatment. NH_3_-N emissions in both fertilized treatments were relatively low after 12 June 2014 (Figure 4I).

Despite differences in N loss, corn yield was statistically equivalent between fertilized treatments and was at least double the unfertilized yield (Table 2). Our results illustrate that significant amounts of N can be lost to the environment in systems using both urea and organic fertilizers, but predominant N loss pathways differ; the organic fertilizer treatment had greater gaseous loss while the urea treatment had greater N leaching (Table 2). A nitrification inhibitor may be advantageous in both systems as it could reduce the quantity of NO_3_-N vulnerable to leaching in the urea treatment and to denitrification in the organic fertilizer treatment.

## Conclusions

Our study illustrates that cover crop species and fertilization schemes affect N loss and availability in corn conservation agriculture systems and that dominant N loss pathways vary by season. During the cover crop growing season, NO_3_-N leaching was the primary loss pathway, especially in treatments using leguminous monocultures, but during the corn growing season, N loss via N_2_O-N and NH_3_-N emissions became dominant, increasing after cover crop termination, fertilizer application, and rain events. Only in the urea N treatment were NO_3_-N leaching losses greater during the corn growing season than during cover crop growth. While fertilizer stimulated corn yield and gaseous N loss vs. unfertilized conditions, fertilizer scheme affected gaseous N loss, temporally and in magnitude, but had limited effects on yield. Similar to conclusions of a recent meta-analysis (Han et al., 2017), our results suggest that employing a legume-grass cover crop mixture and incorporating the N contribution of the cover crop into fertilizer application rates may reduce N loss in conservation agriculture systems without sacrificing yield. Future research should assess whether using these management strategies in combination could further reduce N loss, especially over additional growing seasons, and examine the effect of nitrification inhibitors in similarly managed conventional systems.

## Author contributions

RS, KJ, and RM: Initiated and designed the experiment; RS: Managed the field plots, and collected, processed, and analyzed the data under the mentorship of RM and KJ; RS and RM: Wrote the manuscript and all authors revised the manuscript.

### Conflict of interest statement

The authors declare that the research was conducted in the absence of any commercial or financial relationships that could be construed as a potential conflict of interest.

## Footnotes

^1^FAOSTAT. *Agri-Environmental Indicators/Land*. Available online at: http://faostat3.fao.org/browse/E/EL/E (Accessed November 24, 2015).

^2^Kentucky Mesonet. *Monthly Climatological Summary*. Available online at: http://www.kymesonet.org/historical_data.php (Accessed May 19, 2015).
