# Supplementary material for: Cover Crops and Fertilization Alter Nitrogen Loss in Organic and Conventional Conservation Agriculture Systems
Source: Front Plant Sci. 2018 Jan 22;8:2260. doi: 10.3389/fpls.2017.02260 (PMC5786564; doi:10.3389/fpls.2017.02260)
Supplement: Supplementary file 1 [file SupplementaryMaterials.docx]

Supplementary Material

“Cover crops and fertilization alter nitrogen dynamics in organic and conventional conservation agriculture systems”

R.E. Shelton*, K.L. Jacobsen, R.L. McCulley

*** Correspondence:** Corresponding Author: shelton.rebecca.e@gmail.com

# Supplementary Tables & Figures

Please find included two additional tables and three figures relating to our work, presented in the order in which they appear in the referenced manuscript.

**Supplemental Table S1:** Average (± SE) soil nutrients, texture, structure, and pH of the organic and conventional fields prior to treatment establishment. Methods are as reported in Shelton (2015). Means followed by different letters indicate significant difference within a column (p<0.05).

2014

| **Field** | **Soil-Water pH** | **P** | **K** | **Ca** | **Mg** | **Zn** | **Total N %** | **Total C %** | **Organic Matter %** | **Stable Aggregate Fraction**  **%** | **Bulk Density**  **(g cm^3^)** | **Soil Texture** | | |
| --- | --- | --- | --- | --- | --- | --- | --- | --- | --- | --- | --- | --- | --- | --- |
|  |  | **kg ha^-1^** | | | | |  |  |  |  |  | **Sand %** | **Silt**  **%** | **Clay %** |
| **Organic** | 6.84^a^ (±0.06) | 167.00^a^ (±3.80) | 493.11^a^ (±12.44) | 4090.00^a^ (±115.83) | 354.80^a^  (±4.39) | 5.38^a^ (±0.34) | 0.14^b^ (±0.00) | 1.25^b^ (±0.04) | 2.16^b^  (± 0.07) | 74.49^a^  (**±**3.13) | 1.35^a^  (±0.02) | 8.87^b^ (± 0.19) | 72.82^a^ (±0.31) | 18.31^a^ (±0.31) |
|  | | | | | | | | | | | | | | |
| **Conventional** | 6.33^b^ (±0.08) | 164.33^a^ (±4.91) | 509.11^a^ (±16.06) | 3833.33^a^ (±149.54) | 287.56^b^ (±5.66) | 4.57^b^ (±0.43) | 0.18^a^ (±0.00) | 1.79^a^ (±0.05) | 3.08^a^ (±0.09) | 81.81^a^  (**±**2.67**)** | 1.35^a^  (±0.01) | 10.66^a^ (±0.24) | 72.75^a^ (±0.40) | 16.59^b^ (±0.40) |

Supplemental Fig. S1: Schematic of organic and conventional treatments. For the cover crop comparison, cover crops (vetch, wheat, or a mixture of the two – ‘bi-culture’) were planted in October 2013 and were terminated 20 May 2014. For the organic N-fertilization scheme comparison, a corn crop was planted with or without fertilizer on 28 May 2014 and harvested 6 October 2014. The quantities of applied fertilizer were added in addition to the pre-existing N content of the cover crop. The same hairy vetch, unfertilized (0 fertilizer N) plots were used for the cover crop and fertilizer scheme comparisons in the organic system. For the conventional fertilization scheme comparison, corn was planted following a wheat cover crop and either urea or organic N supplied the full N amount applied.

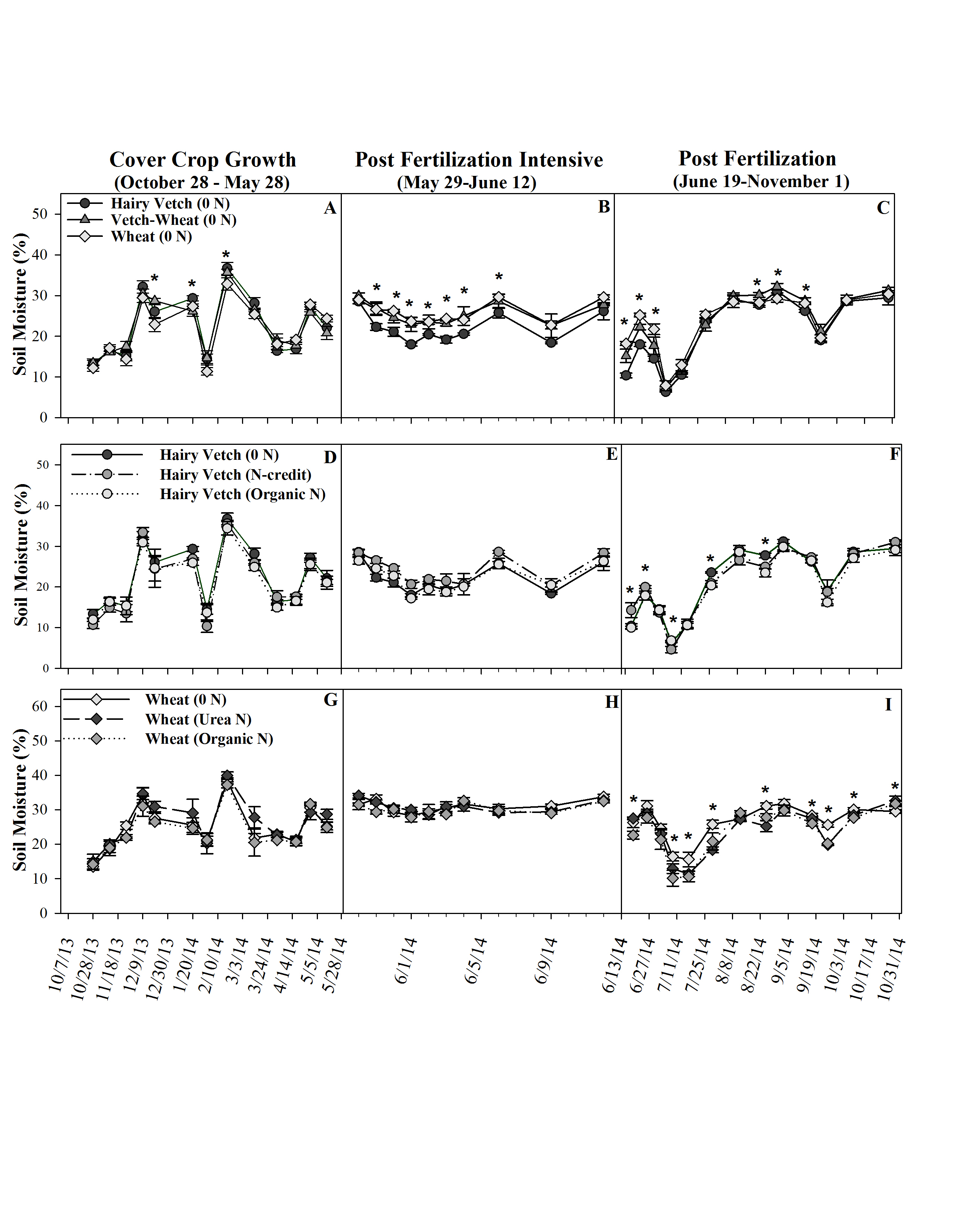
Supplemental Fig. S2: Average soil moisture (±SE) at 5 cm depth for organic cover crop treatments (A-C), organic N-fertilizer treatments (D-F), and conventional N-fertilizer treatments (G-I) across three different time periods from October 2013 to November 2014. Asterisks indicate points in time where significant effects between treatments were observed during the measurement period. Cover crop species are indicated in the legends, where 0 N = no applied fertilizer, N-credit= 56 kg ha^-1^ organic fertilizer + additional hairy vetch, Organic N = 168 kg ha^-1^ organic fertilizer, Urea N = 168 kg ha^-1^ urea + urease inhibitor.


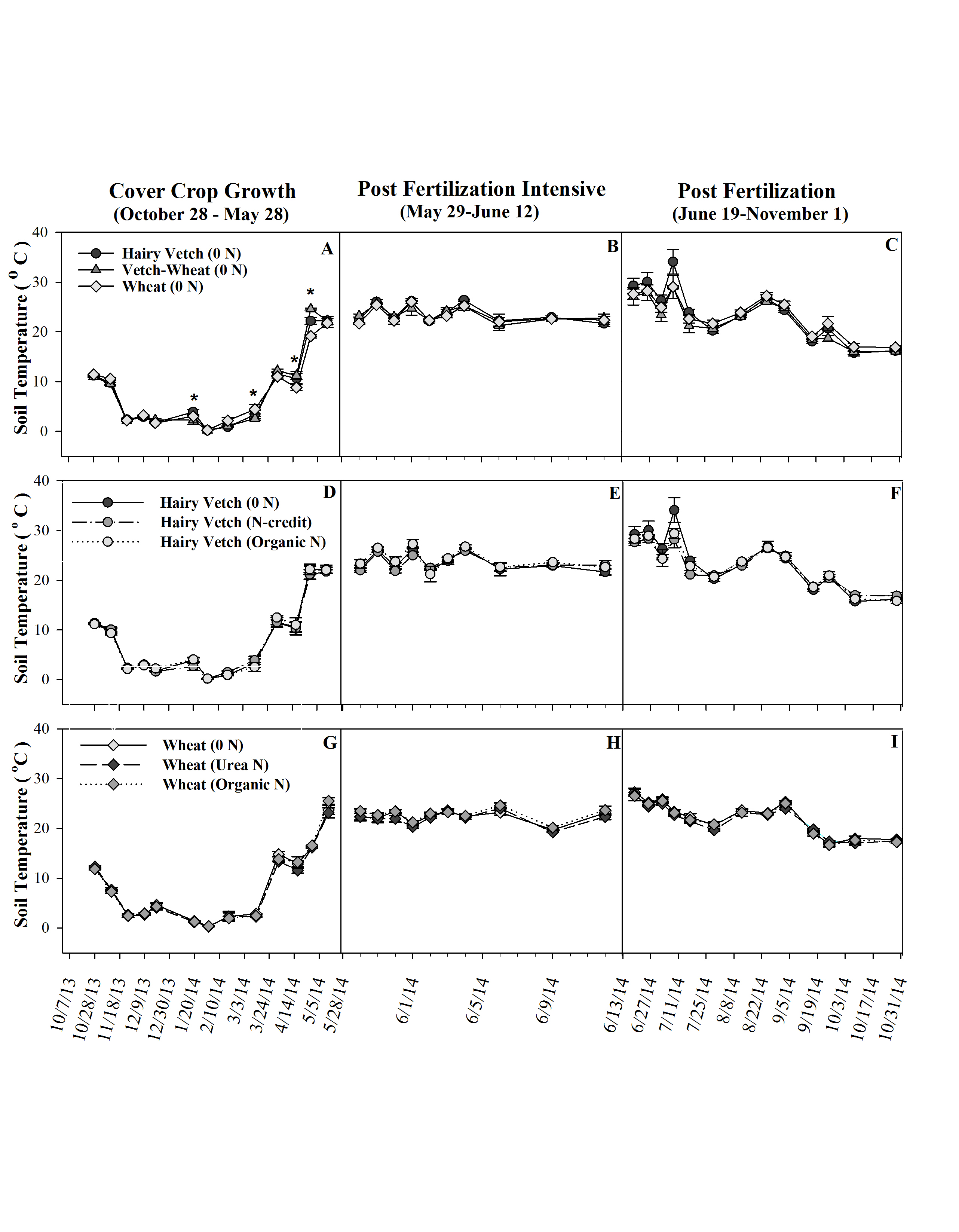


Supplemental Fig. S3: Average soil temperature (±SE) at 5 cm depth for organic cover crop treatments (A-C), organic N-fertilizer treatments (D-F), and conventional N-fertilizer treatments (G-I) across three different time periods from October 2013 to November 2014. Asterisks indicate points in time where significant effects between treatments were observed during the measurement period. Cover crop species are indicated in the legends, where 0 N = no applied fertilizer, N-credit= 56 kg ha^-1^ organic fertilizer + additional hairy vetch, Organic N = 168 kg ha^-1^ organic fertilizer, Urea N = 168 kg ha^-1^ urea + urease inhibitor.
